# Genome-Wide Characterisation of Gene Expression in Rice Leaf Blades at 25°C and 30°C

**DOI:** 10.1155/2014/917292

**Published:** 2014-01-29

**Authors:** Zhi-guo E, Lei Wang, Ryan Qin, Haihong Shen, Jianhua Zhou

**Affiliations:** ^1^China National Rice Research Institute, No. 359, Tiyuchang Road, Hangzhou 310006, China; ^2^iBioinfo Group, Lexington, MA 02421, USA; ^3^School of Life Science, Gwangju Institute of Science and Technology, Gwangju 500-712, Republic of Korea; ^4^Nantong University, Nantong 226001, China

## Abstract

Rice growth is greatly affected by temperature. To examine how temperature influences gene expression in rice on a genome-wide basis, we utilised recently compiled next-generation sequencing datasets and characterised a number of RNA-sequence transcriptome samples in rice seedling leaf blades at 25°C and 30°C. Our analysis indicated that 50.4% of all genes in the rice genome (28,296/56,143) were expressed in rice samples grown at 25°C, whereas slightly fewer genes (50.2%; 28,189/56,143) were expressed in rice leaf blades grown at 30°C. Among the genes that were expressed, approximately 3% were highly expressed, whereas approximately 65% had low levels of expression. Further examination demonstrated that 821 genes had a twofold or higher increase in expression and that 553 genes had a twofold or greater decrease in expression at 25°C. Gene ontology (GO) and Kyoto Encyclopedia of Genes and Genomes (KEGG) analyses suggested that the ribosome pathway and multiple metabolic pathways were upregulated at 25°C. Based on these results, we deduced that gene expression at both transcriptional and translational levels was stimulated at 25°C, perhaps in response to a suboptimal temperature condition. Finally, we observed that temperature markedly regulates several super-families of transcription factors, including bZIP, MYB, and WRKY.

## 1. Introduction

Rice (*Oryza sativa* L.) includes two major subspecies, *indica* and *japonica*. Owing to its importance in food security, extensive studies utilising genetic manipulation, improved cultivation and crossing of subspecies have been conducted in rice during recent decades to improve quality and yield. Like all other plants, rice constantly experiences environmental changes and hostile abiotic stress conditions, such as drought, cold, pollution due to heavy metals and salinity, in addition to biotic stresses, such as viral infection. To minimise abiotic and/or biotic stress-induced damage, plants have developed adaptations and stress tolerance during evolution through the regulation of gene expression and changes in cellular processes. Two major pathways, abscisic acid (ABA)-dependent and ABA-independent pathways, have been extensively studied in plants in response to biotic and/or abiotic stresses [1–3]. ABA is a hormone produced during metabolic reactions. In response to abiotic stress, the ABA-dependent pathway induces the expression of many stress-related genes by regulating the activities of transcription factors. Among many transcription factors, several super-families, including basic leucine zipper (bZIP) [4–6], MYB [7, 8], and WRKY [9–11], have been shown to play critical roles in the regulation of stress response genes in rice.

bZIP proteins include a family of transcriptional regulators that are exclusively present in eukaryotes. Furthermore, they characteristically harbour a bZIP domain composed of two structural features: a DNA-binding basic region and the leucine-zipper dimerisation region. They have been shown to regulate diverse plant-specific phenomena, including seed maturation and germination, flower induction and development, photomorphogenesis and stress and hormone signalling. There are approximately 90 bZIP transcription factor-encoding genes in the rice genome [5].

The *MYB* gene family includes at least 155 members that have been identified by a genome-wide analysis and represents one of the richest groups of transcription factors in rice. MYB proteins are characterised by a highly conserved MYB DNA-binding domain and can be classified into four major groups, 1R-MYB, 2R-MYB, 3R-MYB, and 4R-MYB, on the basis of the number and position of MYB repeats. MYB transcription factors are involved in plant development, secondary metabolism, hormone signal transduction, disease resistance, and abiotic stress tolerance [12].

*WRKY* genes encode transcription factors with a WRKY domain that belongs to zinc-finger proteins. WRKY proteins contain one or two conserved WRKY domains, which are encoded by approximately 60 N-terminal amino acid residues with a WRKYGQ(K/E)K sequence, followed by a C2H2 or C2HC zinc-finger motif. An exhaustive search for *WRKY* genes using HMMER and a hidden Markov model resulted in the identification of 98 and 102 *WRKY* genes in *O. japonica* and *O. indica* rice, respectively. WRKY genes play important roles in disease resistance, responses to salicylic and jasmonic acid, seed development and germination, senescence, abiotic stress responses and ABA responses in rice [13].

Despite all this knowledge, the mechanisms that regulate gene expression in rice are not completely understood. To investigate how external factors, such as temperature, affect rice development and growth through the regulation of gene expression, we searched the available transcriptome databases. We identified two transcriptome RNA-sequence (RNA-Seq) datasets of high quality from rice seedling leaf blades leaf blades grown at 25°C or 30°C. We found that the expression of more than 1300 genes in rice showed a twofold or higher difference between leaf blades that were grown at 25°C compared with those grown at 30°C. Gene ontology (GO) and Kyoto Encyclopedia of Genes and Genomes (KEGG) analyses showed that transcription of many abiotic stress genes and genes involved in ribosome biogenesis were induced at 25°C, indicating that rice grown at 25°C has more active transcription and translation than rice grown at 30°C. Furthermore, we found that among the transcription factor super-families, bZIP, MYB, NAC, and WRKY were significantly regulated in rice at 25°C. Our studies provide useful information on the rice transcriptome in response to suboptimal temperatures.

## 2. Materials and Methods

### 2.1. Transcriptome Sequencing Datasets of Rice Seedling Leaf Blades

Two publicly available RNA-Seq datasets using deep-sequencing of rice seedling leaf blades were downloaded from Gene Expression Omnibus (GEO) under the accession number GSE42096 (http://www.ncbi.nlm.nih.gov/geo/query/acc.cgi?acc=GSE42096) and used for primary analyses. The leaf blades analysed were obtained from wild-type seedlings grown at 30°C or 25°C. For each dataset, RNA-Seq was conducted by paired-end approaches using an Illumina HiSeq 2000 instrument. The read length was 90 bp.

### 2.2. Sequencing Analysis

Sequence alignment between the transcriptome reads was conducted and reads were checked for quality and mapped to the reference genome sequences by Bowtie 2 using the parameters “end-to-end” and “very-sensitive”. The reference genome, transcript annotation, and GO datasets were downloaded from MSU Rice Genome Annotation Project, release 7. The number of reads for a gene was designated as reads per kb per million total reads (RPKM) after normalisation to the number of mapped genome locations. KEGG gene classifications were downloaded from its database.

### 2.3. Statistical Analyses

To determine whether expression was differentially regulated under different temperatures (25°C versus 30°C), we conducted statistical analyses based on the fold-changes in gene expression by adding median counts as a pseudocount. Pathway analyses were based on the binomial probability of observing a number of gene changes in a given pathway. Differences were considered statistically significant when the *P* value was <0.05.

## 3. Results

### 3.1. RNA-Seq Datasets of the Transcriptome from Rice

To accurately determine rice gene expression profiles, we took advantage of recent advances in deep-sequencing technologies. Many RNA-Seq datasets of the transcriptome of rice and other plants are publicly available. Using these datasets, we identified transcriptome sequencing libraries of two rice samples in a single GEO dataset, GSE42096, generated by the Chinese Academy of Sciences and the National Centre for Plant Gene Research (http://www.ncbi.nlm.nih.gov/geo/query/acc.cgi?acc=GSE42096). Profiling of these transcriptome RNAs by high-throughput sequencing was conducted by both single-end and paired-end approaches using an Illumina HiSeq 2000 platform. Paired-end sequencing provided 90 basepairs (bp) per read from each end. RNA-Seq of the transcriptome for each sample generated approximately 25 million reads. Sequence analysis indicated that the datasets were of exceptionally high quality with very low background noise (Supplementary Figure  1 available online on http://dx.doi.org/10.1155/2014/917292).

### 3.2. Gene Expression in Rice at 25°C and 30°C

To characterize expressed transcripts, a reference genome dataset was required. We searched existing databases and found that the MSU Rice Genome Annotation Project on *O. sativa japonica*, released on 31st Oct 2011, is the most complete rice genome database available with more than 56,143 annotated genes, slightly more than the 55986 reported by Kawahara et al. [14]. Therefore, we used this database as our reference for analysing gene expression in the two leaf blade transcriptome datasets. We used Bowtie 2 to align and map the transcriptome reads to rice genome. On the basis of our analysis, we calculated the number of reads as reads per billion (RPB) for each mRNA in two samples. We normalised RPB by read per kbp and RPKM. We found that at 25°C there were 19,766 annotated genes with >1 RPKM read, whereas at 30°C the number of genes that had at least 1 RPKM was 19,350. The distribution of expressed genes with different RPKM levels was similar between 25°C and 30°C (Supplementary Figure 2). Approximately 30 genes had extremely high expression levels (>1000 RPKM), 3% of annotated genes (550–600) were highly expressed (>100 RPKM) and more than 33% of expressed genes (6900) were expressed at only a modest level (10–100 RPKM). Of the genes that were expressed, 64% (12,000) had fewer than 9 RPKM, suggesting that most genes were expressed at a low level. Expression of the other 36,000 genes was not detectable.

### 3.3. Differential Gene Expression in Rice between 30°C and 25°C

We then compared the genes with varied expression in rice growing at 30°C and 25°C. We calculated ratios between the number of reads at 30°C and the number of reads at 25°C. As shown in Supplementary Figure 3, the left part of the histogram shows the number of genes with an increased expression at 25°C. We found that the expression of 257 genes was upregulated more than threefold in rice grown at 25°C, whereas expression of 173 genes was downregulated more than threefold at 25°C. Moreover, there were more genes that were upregulated (564) than downregulated (380) at 25°C, with expression level changes between twofold and threefold. Among approximately 2712 genes with a 1.5–2.0-fold change in expression, 1617 genes were upregulated and 1095 genes (1.5–2.0-fold) were downregulated. Our results indicate that more genes were upregulated at 25°C than at 30°C, suggesting that at 25°C, rice plants need to respond to a suboptimal lower temperature by altering gene transcription.

### 3.4. GO Analysis of Genes That Are Upregulated and Downregulated

To examine the mechanisms of the molecular and cellular responses to a suboptimal lower temperature of 25°C, we performed GO and KEGG analysis in genes of the rice transcriptome that had an twofold or higher change in expression between 25°C and 30°C. In total, we found that the expression of 821 genes was upregulated by twofold or higher at 25°C, whereas 553 genes showed downregulated expression at 25°C. Among the 1374 genes that were either upregulated or downregulated, GO analysis indicated that 4 of the 10 top-ranked GO categories were stress related, with 17.54% of genes (241/1374) related to “response to stress” (ranked at no. 2 with *P* = 1.88*E* − 46), 12.88% of genes (177/1374) related to “response to abiotic stimulus” (ranked at no. 3 with *P* = 1.55*E* − 44), 8.59% of genes (118/1374) related to “response to endogenous stimulus” (ranked at no. 8 with *P* = 4.90*E* − 29) and 6.99% of genes (96/1374) related to “response to biotic stimulus” (ranked at no. 10 with *P* = 1.91*E* − 27), suggesting that 25°C could be considered as a cold-stress condition (Table 1). Other high-ranked GO categories included membrane processes (ranked no. 1) and metabolic processes and ribosome (ranked no. 11), indicating that metabolism and protein translation are perhaps also upregulated at 25°C.

### 3.5. KEGG Pathway Analysis of Upregulated and Downregulated Genes

To further characterise the pathways that are involved in temperature-induced stress responses, we performed KEGG pathway analysis. We observed that the expression of genes that are involved in ribosome biogenesis was significantly upregulated at 25°C, with an adjusted *P* value of 5.2*E* − 29 (Table 2). In addition, 57 of the potential 362 transcripts that had been annotated in the ribosome pathway had a twofold or higher increase in expression at 25°C compared with 30°C. These upregulated transcripts represent 15.7% of the genes in the ribosome pathway. In contrast, none of the 362 transcripts showed decreased expression. Taken together, these results strongly suggested that both transcription and translation were more active at 25°C than 30°C. Other major pathways showing significant changes in expression on KEGG analysis included metabolic and biosynthesis pathways. Of note, *P* values for pathways that were upregulated were markedly more significant than pathways that were downregulated.

### 3.6. Expression Analysis of bZIP, WRKY and MYB Transcription Factors

**Figure 1:**
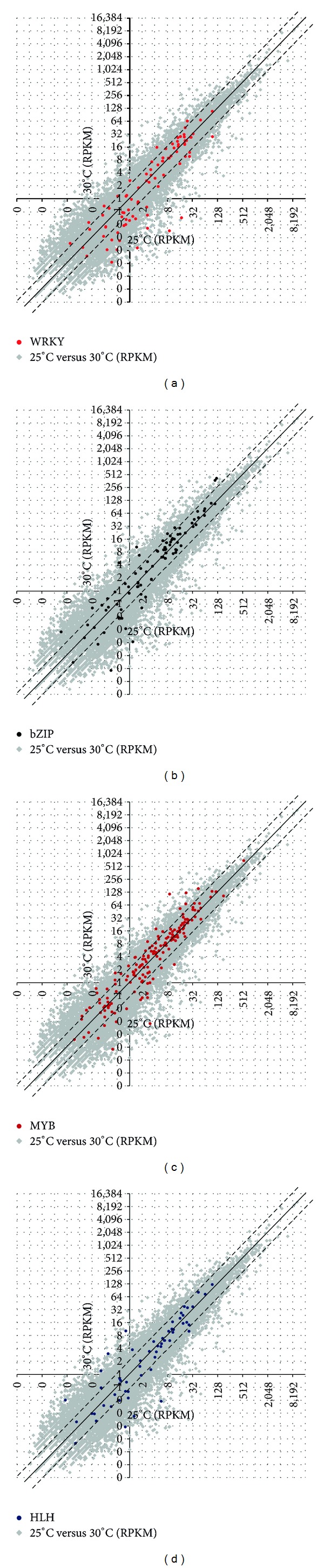
A scatter plot of gene expression values (per kb per million total reads, RPKM) in log2 scale for the 25°C versus 30°C datasets. Grey dots indicate each gene expressed in the two samples. Coloured dots indicate a specific group of genes. Two diagonal dashed lines indicate twofold changes (increases and decreases).

The relative growth rate (RGR) in rice is influenced by temperature, with an optimal growth rate at 30°C [15]. Rice also has a stress response mechanism that is triggered in response to lower temperatures. As described above, transcription is more active at 25°C than at 30°C, indicating that gene expression is stimulated at 25°C. To characterise transcription factors that may be involved in the regulation of gene expression in rice growth at different temperatures and to understand how rice responds to the suboptimal temperature of 25°C, we analysed transcription factor families in rice (Supplementary Table S1), including the expression distribution patterns of the bZIP, MYB, WRKY, and HLH transcription factor super-families (Table 3, Supplementary Table S1 and Figure 1). Approximately 9.5% of bZIP (9/95), 14.1% of WRKY (15/107), 7.8% of MYB (12/128) and 3.5% of HLH (5/145) transcription factors had a twofold or higher change in expression. In contrast, a random calculation suggested that <2.57% (1374/56,143) of the genes should be upregulated or downregulated by twofold or more. Therefore, we conclude that expression of bZIP, WRKY, and MYB super-families was significantly regulated by temperature, with *P* values of 4.96*E* − 08 (WRKY), 4.3*E* − 04 (bZIP) and 0.008 (MYB) between 25°C and 30°C. These data are consistent with previous reports [16, 17] showing that these transcription factor super-families are upregulated or downregulated under colder or warmer temperatures. Other highly regulated transcription factor super-families include NAC and AP2-EREBP. In contrast, other transcription factors such as bHLH and HB did not exhibit significant changes in expression.

## 4. Discussion

Like all plants, rice has to endure constant environmental changes. Among many factors, temperature has been shown to greatly influence rice growth. Rice can grow at a range of temperature, from as low as 12°C to as high as 40°C, but its optimal growth temperature is 30°C or warmer [15]. Unlike mammals, which have a constant body temperature, rice grows at temperatures that fluctuate daily between night and day. One of the mechanisms by which rice can adjust to temperature changes is through regulation of gene expression. Extensive studies have been conducted in rice to analyse the molecular basis of adaptation to both warmer temperatures and cold-stress conditions [3, 18–24]. However, global surveys of temperature-dependent changes in rice gene expression, particularly studies using next generation sequencing technology, are not extensive.

Because the rapid development and reduced cost of both next-generation sequencing and microarray technology, researchers have regularly deposited RNA-Seq datasets for many organisms, including rice, in public domains. For example, several recent papers reported gene expression and splicing in rice using NGS and bioinformatics analysis [25–28]. To investigate how temperature may influence rice growth by affecting gene expression, we searched GEO databases (http://www.ncbi.nlm.nih.gov/geo/). We identified four sets of RNA-Seq transcriptome data from rice (GSE42096, GSE39307, GSE30490, and GSE27240) that were of a high quality. However, among these datasets, only four samples in the GSE42096 dataset, which includes wild-type and TOG1 mutant rice leaf blades, were related to different temperatures (25°C and 30°C). Because mutant TOG1 has not yet been fully described in a public domain, we analysed only two wild-type rice leaf blade samples that grew at both 25°C and 30°C. We realised that the number of samples (two) was limited, but because the quality of these RNA-Seq datasets was exceptionally high, with more than 25 M reads each sample, we believe that our analysis will provide useful information on gene expression in rice to complement similar studies.

Gene expression is regulated at multiple levels. The most fundamental regulatory mechanisms that control the amount of proteins produced are transcription and translation. The *cis*-sequences in a gene, in particular the promoter, and transcription factors dictate how much RNA is transcribed from a gene, whereas ribosomes are directly related to the activities of protein translation. In our investigation, we demonstrated that the number of the genes that are expressed at 30°C (17,356) is similar to that at 25°C (17,966), indicating that it is necessary for only 1/3 of rice genes to be expressed to maintain growth under a given condition. Similarly, we observed that about 3% of genes were highly expressed at both 30°C and 25°C. The rest of the expressed genes had either a modest or a low expression level or no detectable expression.

However, the difference lies in the expression levels of specific genes between samples at 25°C and 30°C. We showed that 3986 genes had either increased or decreased expression levels of 1.50-fold or higher. Among these 3986 genes, 1374 had a twofold or higher increase (821) or decrease (553) when the temperature dropped from 30°C to 25°C, indicating perhaps that rice at 25°C has more active transcription. Consistent with this notion, by GO and KEGG analyses, we found that a significant number of genes in the ribosome pathways were upregulated at 25°C, suggesting that translation may be also more robust at 25°C. Considering previous reports that rice has a better growth rate at 30°C, or at least at temperatures warmer than 25°C [15], the more active transcription and translation at 25°C can be explained by the response of rice to a colder temperature. In fact, we observed in our GO analysis that 4 of the 12 top-ranked pathway categories were related to stress response. We deduced from our results that although 30°C is an optimal temperature for rice growth, transcription and translation for many genes are triggered at 25°C and this temperature (25°C) may be minimally sufficient to trigger the cold stress-response.

To further examine the molecular basis of the differential expression of rice genes between 25°C and 30°C, we examined rice transcription factor families (Figure 1, Table 3, Supplementary Table S1). In contrast to the WRKY super-family, bZIP and MYB transcription factor super-families had more downregulated than upregulated genes (1.5-fold cutoff) at 25°C. Although we were unable to draw definitive conclusions from this analysis, our results suggest that the WRKY super-family plays a positive role in the response to lower temperatures, whereas both bZIP and MYB super-families may have a negative impact on gene expression at 25°C, in agreement with a previous report that bZIP transcription factors, such as ZIP52, are negative regulators of cold stress [5]. In addition, we also showed that many transcription factors, including bHLH and HB, are not significantly regulated between 30°C and 25°C.

## 5. Conclusion

We concluded that only a small percentage of genes (3%) have a high expression level whereas more than 60% of genes have a very low expression level in rice. Both transcription and translation are more active at 25°C than at 30°C. Expression of bZIP, WRKY, and MYB is significantly regulated at 25°C.

## Supplementary Material

Supplementary Figure 1. Datasets quality chart. SRR611648 and SRR611649 are two RNA-seq transcriptome datasets from rice leaf blades at 25°C or 30°C that are identified from Gene Expression Omnibus (GEO) under the accession number GSE42096. Ninety quality score bar charts align on the x axis, one for each base pair of read. The Y axis is the quality sore. The yellow box in the bar chart is the area between quartile 1 and quartile 3. The black line is the mean line and the short horizontal red line is the median.
Supplementary Figure 2. A histogram showing the distribution patterns of gene expression in RNA-seq transcriptome samples in rice. Log2(RMKM) represents the number of RPKM (Log2) obtained from analysis of RNA-seq datasets. Frequency represents the number of genes with a defined expression level (RPKM). Red: 25°C; green: 30°C; orange: overlap between 25°C and 30°C.
Supplementary Figure 3. A histogram showing differential expression of genes in rice leaf blades at 25°C and 30°C. “Log2(fold change) 30°C/25°C” represents the number of fold (Log2) of gene expression that is regulated from 25°C to 30°C; “Log(frequency)” represents the number of genes with a defined fold of change in gene expression from 30°C to 25°C. Left panel: the number of genes with increased expression at 25°C. Right panel: the number of genes with increased expression at 30°C.
Table S1: Expressed genes in rice leaf blades at 250C and 300C. Gene: Annotated gene ID; Exon-length: length of the exon; Location: chromosome location; Description: gene description; SRR611648(RPKM): the number of reads at 250C; SRR611649(RPKM): the number of reads at 300C; SRR611648(RPKM+median): the adjusted number of reads at 250C; SRR611649(RPKM+median): the adjusted number of reads at 300C; fldchg(WT): ratio between SRR611649(RPKM+median)/ SRR611648(RPKM+median).

## Figures and Tables

**Table 1:** Top ranked pathways that are regulated (1374 genes) at 25°C by GO analysis.

Rank	Pathway	Pathway annotation	Pathway size	Observed	Ratio	*P* value
1	Process: metabolic process	rice:GO:0008152	7390	391	0.05	4.3*E* − 52
2	Process: response to stress	rice:GO:0006950	3620	241	0.07	1.9*E* − 46
3	Process: response to abiotic stimulus	rice:GO:0009628	2195	177	0.08	1.5*E* − 44
4	Process: cellular process	rice:GO:0009987	7325	365	0.05	3.3*E* − 42
5	Function: catalytic activity	rice:GO:0003824	3688	227	0.06	2.7*E* − 38
6	Component: membrane	rice:GO:0016020	3728	220	0.06	2.1*E* − 34
7	Process: biosynthetic process	rice:GO:0009058	4673	250	0.05	1.6*E* − 32
8	Process: response to endogenous stimulus	rice:GO:0009719	1490	118	0.08	4.9*E* − 29
9	Component: cytosol	rice:GO:0005829	2289	151	0.07	2.1*E* − 28
10	Process: response to biotic stimulus	rice:GO:0009607	1081	96	0.09	1.9*E* − 27
11	Component: ribosome	rice:GO:0005840	481	61	0.13	9.5*E* − 26
12	Function: structural molecule activity	rice:GO:0005198	518	63	0.12	1.5*E* − 25

**Table 2:** Top ranked pathways that are regulated (1374 genes) at 25°C by KEGG analysis.

Rank	Pathway	Pathway annotation	Pathway size	Observed	Ratio	*P* value
1	Ribosome	rice:osa03010	362	57	0.16	5.2*E* − 29
2	Metabolic pathways	rice:osa01100	1565	119	0.08	1.0*E* − 27
3	Biosynthesis of secondary metabolites	rice:osa01110	745	65	0.09	1.3*E* − 18
4	Starch and sucrose metabolism	rice:osa00500	130	20	0.15	5.9*E* − 11
5	Alpha-Linolenic acid metabolism	rice:osa00592	34	9	0.26	8.7*E* − 08
6	Glyoxylate and dicarboxylate metabolism	rice:osa00630	62	11	0.18	2.6*E* − 7
7	Carbon fixation in photosynthetic organisms	rice:osa00710	85	12	0.14	9.6*E* − 07
8	Diterpenoid biosynthesis	rice:osa00904	24	7	0.29	1.2*E* − 06
9	Photosynthesis	rice:osa00195	148	15	0.10	3.2*E* − 06
10	Plant hormone signal transduction	rice:osa04075	150	14	0.09	1.6*E* − 05
11	Biosynthesis of unsaturated fatty acids	rice:osa01040	44	7	0.16	8.0*E* − 05
12	Phenylpropanoid biosynthesis	rice:osa00940	94	9	0.10	4.0*E* − 04

**Table 3:** Top ranked transcription factors that are regulated in rice by temperature.

Rank	Pathway	Pathway annotation	Pathway size	Observed	Ratio	*P* value
1	Rice transcription factor: WRKY	rice:TF:WRKY	107	15	0.14	4.9*E* − 08
2	Rice transcription factor: NAC	rice:TF:NAC	124	12	0.096	4.5*E* − 05
3	Rice transcription factor: AP2-EREBP	rice:TF:AP2-EREBP	169	14	0.082	5.9*E* − 05
4	Rice transcription factor: orphans	rice:TF:orphans	85	9	0.105	0.00019
5	Rice transcription factor: bZIP	rice:TF:bZIP	95	9	0.095	0.00043
6	Rice transcription factor: MYB	rice:TF:MYB	128	10	0.078	0.00092
7	Rice transcription factor: tify	rice:TF:tify	21	4	0.190	0.00140
8	Rice transcription factor: MYB-related	rice:TF:MYB-related	100	7	0.07	0.00835
9	Rice transcription factor: C2H2	rice:TF:C2H2	104	7	0.067	0.01004
10	Rice transcription factor: pseudo ARR-B	rice:TF:pseudo_ARR-B	9	2	0.222	0.01811
11	Rice transcription factor: G2-like	rice:TF:G2-like	48	4	0.083	0.02342
12	Rice transcription factor: WRKY	rice:TF:WRKY	107	15	0.140	4.95634
