# Supplementary material for: Genome-Wide Characterisation of Gene Expression in Rice Leaf Blades at 25°C and 30°C
Source: ScientificWorldJournal. 2014 Jan 29;2014:917292. doi: 10.1155/2014/917292 (PMC3929188; doi:10.1155/2014/917292)
Supplement: Supplementary file 1 — Supplementary Figure 1. Datasets quality chart. SRR611648 and SRR611649 are two RNA-seq transcriptome datasets from rice leaf blades at 25°C or 30°C that are identified from Gene Expression Omnibus (GEO) under the accession number GSE42096. Ninety quality score bar charts align on the x axis, one for each base pair of read. The Y axis is the quality sore. The yellow box in the bar chart is the area between quartile 1 and quartile 3. The black line is the mean line and the short horizontal red line is the median. Supplementary Figure 2. A histogram showing the distribution patterns of gene expression in RNA-seq transcriptome samples in rice. Log2(RMKM) represents the number of RPKM (Log2) obtained from analysis of RNA-seq datasets. Frequency represents the number of genes with a defined expression level (RPKM). Red: 25°C; green: 30°C; orange: overlap between 25°C and 30°C. Supplementary Figure 3. A histogram showing differential expression of genes in rice leaf blades at 25°C and 30°C. “Log2(fold change) 30°C/25°C” represents the number of fold (Log2) of gene expression that is regulated from 25°C to 30°C; “Log(frequency)” represents the number of genes with a defined fold of change in gene expression from 30°C to 25°C. Left panel: the number of genes with increased expression at 25°C. Right panel: the number of genes with increased expression at 30°C. Table S1: Expressed genes in rice leaf blades at 250C and 300C. Gene: Annotated gene ID; Exon-length: length of the exon; Location: chromosome location; Description: gene description; SRR611648(RPKM): the number of reads at 250C; SRR611649(RPKM): the number of reads at 300C; SRR611648(RPKM+median): the adjusted number of reads at 250C; SRR611649(RPKM+median): the adjusted number of reads at 300C; fldchg(WT): ratio between SRR611649(RPKM+median)/ SRR611648(RPKM+median). [file 917292.f1.docx]

**Supplementary Figures**

**Supplementary Figure 1.** Datasets quality chart**.** SRR611648 and SRR611649 are two RNA-seq transcriptome datasets from rice leaf blades at 25°C or 30°C that are identified from Gene Expression Omnibus (GEO) under the accession number GSE42096. Ninety quality score bar charts align on the x axis, one for each base pair of read. The Y axis is the quality sore. The yellow box in the bar chart is the area between quartile 1 and quartile 3. The black line is the mean line and the short horizontal red line is the median.

**Supplementary Figure 2.** A histogram showing the distribution patterns of gene expression in RNA-seq transcriptome samples in rice. Log2(RMKM) represents the number of RPKM (Log2) obtained from analysis of RNA-seq datasets. Frequency represents the number of genes with a defined expression level (RPKM). Red: 25°C; green: 30°C; orange: overlap between 25°C and 30°C.

**Supplementary Figure 3. A** histogram showing differential expression of genes in rice leaf blades at 25°C and 30°C. “Log2(fold change) 30°C/25°C” represents the number of fold (Log2) of gene expression that is regulated from 25°C to 30°C; “Log(frequency)” represents the number of genes with a defined fold of change in gene expression from 30°C to 25°C. Left panel: the number of genes with increased expression at 25°C. Right panel: the number of genes with increased expression at 30°C.

**
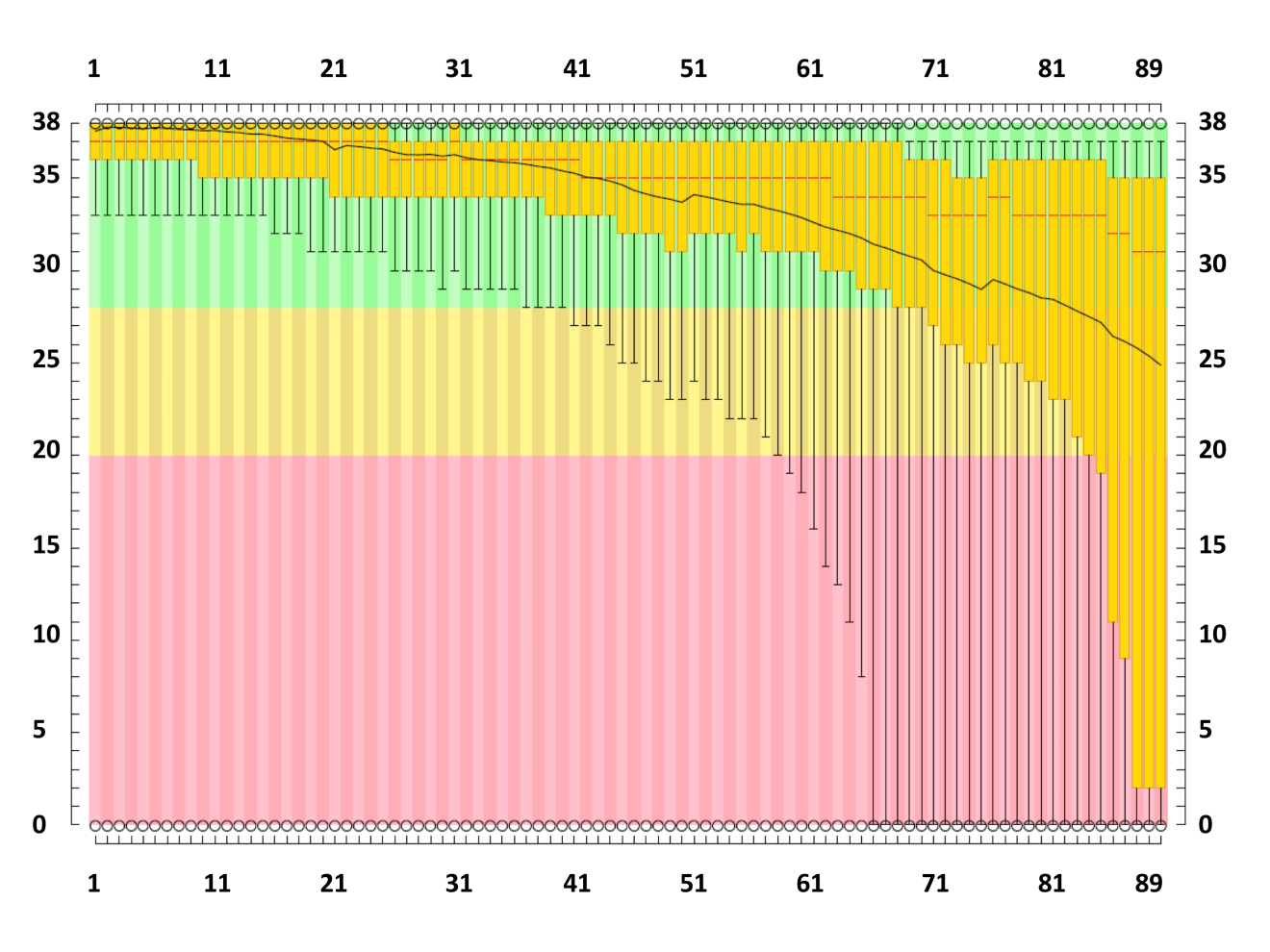
**

**Supplementary Figure 1**


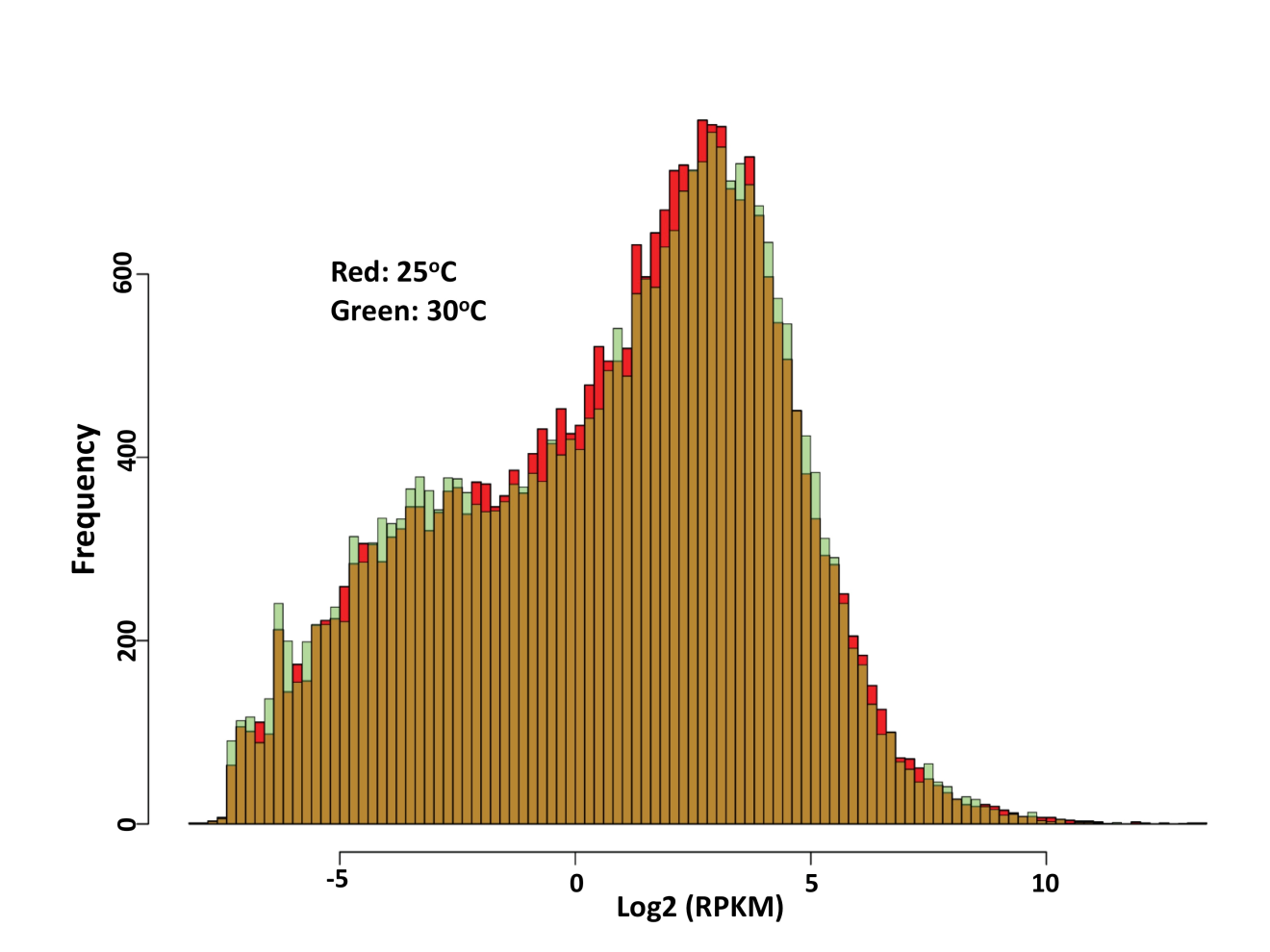


Supplementary Figure 2


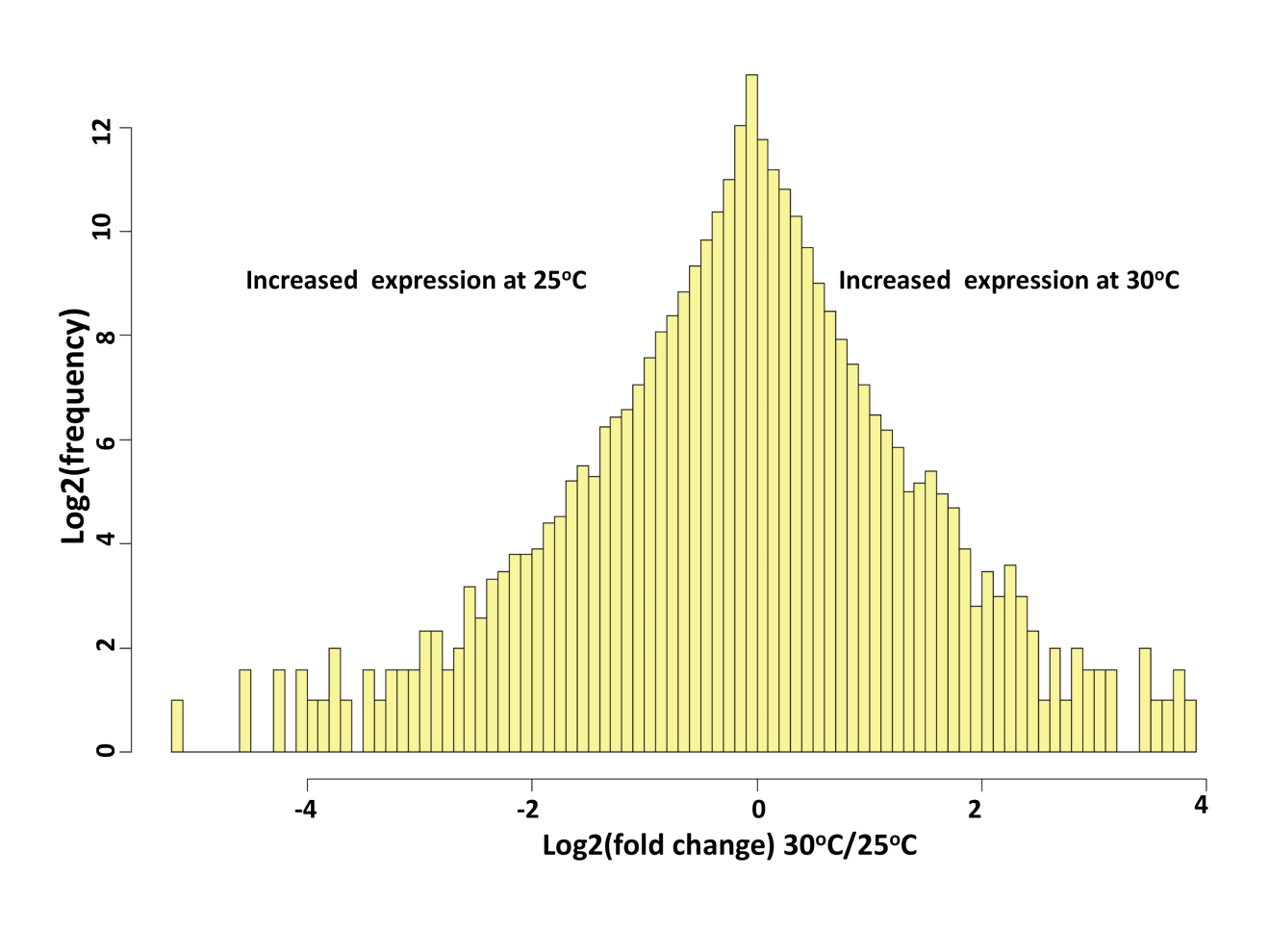


Supplementary Figure 3

**Supplementary Table S1: Regulation of Transcription factors in rice at 25^o^C and 30^o^C.**

| genes involved | 1374 |  |  |  |  |  |  |
| --- | --- | --- | --- | --- | --- | --- | --- |
| Pathway | Observed | Pathway_size | Ratio | P-value | Description | |  |
| rice:TF:WRKY | 15 | 107 | 0.140187 | 4.96E-08 | rice transcription factor: WRKY | | |
| rice:TF:NAC | 12 | 124 | 0.096774 | 4.47E-05 | rice transcription factor: NAC | | |
| rice:TF:AP2-EREBP | 14 | 169 | 0.08284 | 5.89E-05 | rice transcription factor: AP2-EREBP | | |
| rice:TF:Orphans | 9 | 85 | 0.105882 | 0.000194 | rice transcription factor: Orphans | | |
| rice:TF:bZIP | 9 | 95 | 0.094737 | 0.000433 | rice transcription factor: bZIP | | |
| rice:TF:MYB | 10 | 128 | 0.078125 | 0.000926 | rice transcription factor: MYB | | |
| rice:TF:Tify | 4 | 21 | 0.190476 | 0.001405 | rice transcription factor: Tify | | |
| rice:TF:MYB-related | 7 | 100 | 0.07 | 0.008359 | rice transcription factor: MYB-related | | |
| rice:TF:C2H2 | 7 | 104 | 0.067308 | 0.010049 | rice transcription factor: C2H2 | | |
| rice:TF:Pseudo_ARR-B | 2 | 9 | 0.222222 | 0.01812 | rice transcription factor: Pseudo ARR-B | | |
| rice:TF:G2-like | 4 | 48 | 0.083333 | 0.023427 | rice transcription factor: G2-like | | |
| rice:TF:FAR1 | 0 | 126 | 0 | 0.043915 | rice transcription factor: FAR1 | | |
| rice:TF:HSF | 2 | 28 | 0.071429 | 0.118884 | rice transcription factor: HSF | | |
| rice:TF:HB | 4 | 100 | 0.04 | 0.130432 | rice transcription factor: HB | | |
| rice:TF:bHLH | 5 | 145 | 0.034483 | 0.136389 | rice transcription factor: bHLH | | |
| rice:TF:AUX/IAA | 2 | 32 | 0.0625 | 0.14129 | rice transcription factor: AUX/IAA | | |
| rice:TF:mTERF | 2 | 33 | 0.060606 | 0.146728 | rice transcription factor: mTERF | | |
| rice:TF:C3H | 0 | 74 | 0 | 0.159653 | rice transcription factor: C3H | | |
| rice:TF:MADS | 0 | 72 | 0 | 0.167774 | rice transcription factor: MADS | | |
| rice:TF:LOB | 2 | 39 | 0.051282 | 0.177495 | rice transcription factor: LOB | | |
| rice:TF:TRAF | 3 | 80 | 0.0375 | 0.178832 | rice transcription factor: TRAF | | |
| rice:TF:DBP | 1 | 9 | 0.111111 | 0.180677 | rice transcription factor: DBP | | |
| rice:TF:Sigma70-like | 1 | 9 | 0.111111 | 0.180677 | rice transcription factor: Sigma70-like | | |
| rice:TF:BES1 | 1 | 10 | 0.1 | 0.195842 | rice transcription factor: BES1 | | |
| rice:TF:ARR-B | 1 | 12 | 0.083333 | 0.223655 | rice transcription factor: ARR-B | | |
| rice:TF:GRAS | 0 | 60 | 0 | 0.225948 | rice transcription factor: GRAS | | |
| rice:TF:RWP-RK | 1 | 15 | 0.066667 | 0.25955 | rice transcription factor: RWP-RK | | |
| rice:TF:PHD | 0 | 51 | 0 | 0.282456 | rice transcription factor: PHD | | |
| rice:TF:zf-HD | 1 | 18 | 0.055556 | 0.289158 | rice transcription factor: zf-HD | | |
| rice:TF:C2C2-CO-like | 1 | 21 | 0.047619 | 0.313193 | rice transcription factor: C2C2-CO-like | | |
| rice:TF:ABI3VP1 | 1 | 59 | 0.016949 | 0.343198 | rice transcription factor: ABI3VP1 | | |
| rice:TF:C2C2-GATA | 1 | 29 | 0.034483 | 0.354756 | rice transcription factor: C2C2-GATA | | |
| rice:TF:CCAAT | 1 | 49 | 0.020408 | 0.365192 | rice transcription factor: CCAAT | | |
| rice:TF:C2C2-Dof | 1 | 33 | 0.030303 | 0.365604 | rice transcription factor: C2C2-Dof | | |
| rice:TF:OFP | 1 | 35 | 0.028571 | 0.369017 | rice transcription factor: OFP | | |
| rice:TF:SNF2 | 1 | 41 | 0.02439 | 0.372564 | rice transcription factor: SNF2 | | |
| rice:TF:GNAT | 1 | 40 | 0.025 | 0.372596 | rice transcription factor: GNAT | | |
| rice:TF:SET | 0 | 34 | 0 | 0.43055 | rice transcription factor: SET | | |
| rice:TF:ARF | 0 | 28 | 0 | 0.499602 | rice transcription factor: ARF | | |
| rice:TF:TCP | 0 | 26 | 0 | 0.524996 | rice transcription factor: TCP | | |
| rice:TF:Trihelix | 0 | 24 | 0 | 0.55168 | rice transcription factor: Trihelix | | |
| rice:TF:SBP | 0 | 22 | 0 | 0.579719 | rice transcription factor: SBP | | |
| rice:TF:FHA | 0 | 21 | 0 | 0.594268 | rice transcription factor: FHA | | |
| rice:TF:Jumonji | 0 | 18 | 0 | 0.640141 | rice transcription factor: Jumonji | | |
| rice:TF:PLATZ | 0 | 18 | 0 | 0.640141 | rice transcription factor: PLATZ | | |
| rice:TF:TUB | 0 | 17 | 0 | 0.656206 | rice transcription factor: TUB | | |
| rice:TF:GRF | 0 | 15 | 0 | 0.689553 | rice transcription factor: GRF | | |
| rice:TF:CPP | 0 | 14 | 0 | 0.706856 | rice transcription factor: CPP | | |
| rice:TF:SWI/SNF-BAF60b | 0 | 14 | 0 | 0.706856 | rice transcription factor: SWI/SNF-BAF60b | | |
| rice:TF:BSD | 0 | 13 | 0 | 0.724593 | rice transcription factor: BSD | | |
| rice:TF:Alfin-like | 0 | 12 | 0 | 0.742775 | rice transcription factor: Alfin-like | | |
| rice:TF:E2F-DP | 0 | 12 | 0 | 0.742775 | rice transcription factor: E2F-DP | | |
| rice:TF:EIL | 0 | 12 | 0 | 0.742775 | rice transcription factor: EIL | | |
| rice:TF:HMG | 0 | 12 | 0 | 0.742775 | rice transcription factor: HMG | | |
| rice:TF:C2C2-YABBY | 0 | 11 | 0 | 0.761413 | rice transcription factor: C2C2-YABBY | | |
| rice:TF:CAMTA | 0 | 10 | 0 | 0.780519 | rice transcription factor: CAMTA | | |
| rice:TF:DDT | 0 | 9 | 0 | 0.800103 | rice transcription factor: DDT | | |
| rice:TF:GeBP | 0 | 9 | 0 | 0.800103 | rice transcription factor: GeBP | | |
| rice:TF:LIM | 0 | 9 | 0 | 0.800103 | rice transcription factor: LIM | | |
| rice:TF:LUG | 0 | 9 | 0 | 0.800103 | rice transcription factor: LUG | | |
| rice:TF:ARID | 0 | 8 | 0 | 0.820178 | rice transcription factor: ARID | | |
| rice:TF:Rcd1-like | 0 | 8 | 0 | 0.820178 | rice transcription factor: Rcd1-like | | |
| rice:TF:SRS | 0 | 8 | 0 | 0.820178 | rice transcription factor: SRS | | |
| rice:TF:BBR/BPC | 0 | 7 | 0 | 0.840757 | rice transcription factor: BBR/BPC | | |
| rice:TF:Coactivator_p15 | 0 | 7 | 0 | 0.840757 | rice transcription factor: Coactivator p15 | | |
| rice:TF:SWI/SNF-SWI3 | 0 | 7 | 0 | 0.840757 | rice transcription factor: SWI/SNF-SWI3 | | |
| rice:TF:CSD | 0 | 6 | 0 | 0.861851 | rice transcription factor: CSD | | |
| rice:TF:TAZ | 0 | 6 | 0 | 0.861851 | rice transcription factor: TAZ | | |
| rice:TF:MBF1 | 0 | 5 | 0 | 0.883475 | rice transcription factor: MBF1 | | |
| rice:TF:PBF-2-like | 0 | 5 | 0 | 0.883475 | rice transcription factor: PBF-2-like | | |
| rice:TF:RB | 0 | 5 | 0 | 0.883475 | rice transcription factor: RB | | |
| rice:TF:S1Fa-like | 0 | 5 | 0 | 0.883475 | rice transcription factor: S1Fa-like | | |
| rice:TF:SOH1 | 0 | 5 | 0 | 0.883475 | rice transcription factor: SOH1 | | |
| rice:TF:ULT | 0 | 5 | 0 | 0.883475 | rice transcription factor: ULT | | |
| rice:TF:VOZ | 0 | 5 | 0 | 0.883475 | rice transcription factor: VOZ | | |
| rice:TF:HRT | 0 | 4 | 0 | 0.90564 | rice transcription factor: HRT | | |
| rice:TF:IWS1 | 0 | 4 | 0 | 0.90564 | rice transcription factor: IWS1 | | |
| rice:TF:LFY | 0 | 4 | 0 | 0.90564 | rice transcription factor: LFY | | |
| rice:TF:MED6 | 0 | 4 | 0 | 0.90564 | rice transcription factor: MED6 | | |
| rice:TF:MED7 | 0 | 4 | 0 | 0.90564 | rice transcription factor: MED7 | | |
